# Longitudinal Risk Analysis of Second Primary Cancer after Curative Treatment in Patients with Rectal Cancer

**DOI:** 10.3390/diagnostics14131461

**Published:** 2024-07-08

**Authors:** Jiun-Yi Hsia, Chi-Chang Chang, Chung-Feng Liu, Chia-Lin Chou, Ching-Chieh Yang

**Affiliations:** 1 Division of Thoracic Surgery, Department of Surgery, Chung Shan Medical University Hospital, Taichung 402367, Taiwan; cshy1700@csh.org.tw; 2 School of Medicine, Chung Shan Medical University, Taichung 40201, Taiwan; 3 School of Medical Informatics, Chung Shan Medical University, IT Office, Chung Shan Medical University Hospital, Taichung 40201, Taiwan; changintw@gmail.com; 4 Department of Information Management, Ming Chuan University, Taoyuan 33348, Taiwan; 5 Department of Medical Research, Chi Mei Medical Center, Tainan 710402, Taiwan; chungfengliu@gmail.com; 6 Division of Colon & Rectal Surgery, Department of Surgery, Chi Mei Medical Center, Tainan 710402, Taiwan; 7 Department of Medical Laboratory Science and Biotechnology, Chung Hwa University of Medical Technology, Tainan 71703, Taiwan; 8 Department of Radiation Oncology, Chi Mei Medical Center, Tainan 71004, Taiwan; 9 Department of Pharmacy, Chia-Nan University of Pharmacy and Science, Tainan 717301, Taiwan; 10 School of Medicine, College of Medicine, National Sun Yat-sen University, Kaohsiung 80404, Taiwan

**Keywords:** second primary cancers, rectal cancer, chemotherapy, clinical decision-making model, machine learning techniques

## Abstract

Predicting and improving the response of rectal cancer to second primary cancers (SPCs) remains an active and challenging field of clinical research. Identifying predictive risk factors for SPCs will help guide more personalized treatment strategies. In this study, we propose that experience data be used as evidence to support patient-oriented decision-making. The proposed model consists of two main components: a pipeline for extraction and classification and a clinical risk assessment. The study includes 4402 patient datasets, including 395 SPC patients, collected from three cancer registry databases at three medical centers; based on literature reviews and discussion with clinical experts, 10 predictive variables were considered risk factors for SPCs. The proposed extraction and classification pipelines that classified patients according to importance were age at diagnosis, chemotherapy, smoking behavior, combined stage group, and sex, as has been proven in previous studies. The C5 method had the highest predicted AUC (84.88%). In addition, the proposed model was associated with a classification pipeline that showed an acceptable testing accuracy of 80.85%, a recall of 79.97%, a specificity of 88.12%, a precision of 85.79%, and an F1 score of 79.88%. Our results indicate that chemotherapy is the most important prognostic risk factor for SPCs in rectal cancer survivors. Furthermore, our decision tree for clinical risk assessment illuminates the possibility of assessing the effectiveness of a combination of these risk factors. This proposed model may provide an essential evaluation and longitudinal change for personalized treatment of rectal cancer survivors in the future.

## 1. Introduction

Rectal cancer is among the most common malignancies, affecting one-third of all colorectal cancer patients worldwide [1]. A multidisciplinary approach to rectal cancer treatment includes preoperative therapy followed by total mesorectum excision and adjuvant chemotherapy [2]. The development of new anticancer regimens, such as monoclonal antibodies and immune checkpoint inhibitors, has significantly decreased the mortality rate of rectal cancer [3,4]. Due to the increased long-term survival of rectal cancer survivors, second primary cancers (SPCs) are receiving increasing attention in clinical practice [5].

Phipps et al. found a higher rate of SPCs among rectal cancer survivors compared to the general population. As a result of various lifestyle, genetic, environmental, and treatment factors, SPCs in rectal cancer survivors are associated with the use of alcohol, tobacco, betel nuts, and anticancer drugs [6,7,8]. Several studies have also examined the effect of radiotherapy or chemotherapy on SPC risk, with inconsistent results [9,10]. Currently, no risk factors have been established that can predict the response to SPCs, and no tools have been incorporated into clinical practice to improve the prediction of SPCs in patients with rectal cancer. The objective of this study was to determine the risk factors for SPCs and perform a clinical assessment of the risk of rectal cancer to ultimately contribute to clinical treatment.

## 2. Materials and Methods 

### 2.1. Ethic Statement

Chi-Mei Medical Center Institutional Review Board (CMFHR11006-006) approved this study in accordance with the Declaration of Helsinki. Because no personally identifiable information was used, the IRB waived the need for individual informed consent. In addition, this study had a noninterventional retrospective design, with no human subjects, and all data were analyzed anonymously.

### 2.2. Study Population

We included 4402 patients diagnosed with rectal cancer across multiple institutions between 1 January 2009 and 31 December 2016. The follow-up deadline was 31 December 2022 for survivors. All samples in this study were classified according to the 7th edition of the American Cancer Committee, and samples were selected considering second primary cancer [11,12,13,14,15,16].

All data were collected based on the following criteria: (1) considering the International Classification of Diseases for Oncology, 3rd edition, cases with the primary site of the rectosigmoid junction (code C19.9) and the rectum (code C20.9); (2) patients treated in the hospital who met the previous criterion. The exclusion criteria were (1) no clear coding on follow-up or curable treatment; (2) never being disease-free; (3) previous cancer history or metastatic disease or missing coding; and (4) SPCs diagnosed within 6 months, which were excluded from this study as we sought to investigate the prevention of recurrence and metastasis to observe the effect of treatment over time. Furthermore, the NCCN’s latest guidelines recommend a 6-month first surveillance examination after the removal of large adenomas or sessile serrated polyps with unfavorable features or those that have been sporadically removed [17].

### 2.3. The Evidence-Based Clinical Decision-Making Model

Three cancer registry databases were used, and coding data collected from three medical centers were input into the model as case data. Then, 10 important risk factors were considered, namely (1) sex, (2) age at diagnosis, (3) tumor size, (4) combined stage group, (5) radiotherapy, (6) chemotherapy, (7) body mass index (BMI) (kg/m^2^), (8) smoking behavior, (9) drinking behavior, and (10) carcinoembryonic antigen (CEA) lab value. To ensure the robustness and accuracy of our predictive models, we implemented a comprehensive validation strategy. Initially, the original dataset was divided into training and testing datasets, with a separation rate of 7:3. This initial split was used to perform a preliminary assessment of the model’s performance, providing a baseline indication of its effectiveness in new, unseen data scenarios. During this initial validation phase, several metrics such as accuracy, sensitivity, specificity, and the area under the receiver operating characteristic curve (AUC) were calculated to evaluate the model’s prediction ability. These metrics helped identify any potential overfitting at an early stage and guided the further tuning of model parameters. Furthermore, to enhance the generalizability of our model, we applied a 10-fold cross-validation technique within the training dataset. During the training period, all training datasets were randomly divided into 10 subsets of equal size, with each subset playing a role in the validation dataset. The format of the test dataset was the same as the training dataset.

In the extraction and classification pipelines, we used two types of extracting processes. One was the machine learning technique, and the other was the statistical testing method. In the machine learning technique, all 10 risk factors were directly used as predictors for C5.0, random forest (RF), C4.5, classification and regression tree (CART), support vector machine (SVM), logistic regression (LGR), and linear discriminant analysis (LDA) for constructing seven classification pipelines. Based on our previous studies, support vector machines classify classes using a linear decision boundary called the hyperplane. Hyperplanes place data to maximize the distance between the instance and the hyperplane [18,19]. Linear discriminant analysis is a supervised learning algorithm that also extracts features and compresses data for downscaling and classification [20,21]. Logistic regression is the most commonly used approach in epidemiology and medicine. A generalized linear model explicitly models the relationship between the explanatory variable X and the response variable Y [22,23]. Based on the concept of information entropy, C4.5 decision trees select the attributes of each node according to their attributes [24]. Using a greedy approach, decision trees were built in a top-down, recursive, and divide-and-conquer manner. In random forests, subsets of the dataset predictor variables are randomly selected, and the results are consolidated to generate a classification tree [25]. Using a recursive process, the C5.0 decision tree generates a tree based on the provided information using a top-down approach [26]. For splitting and estimation, the Gini index was used to construct the classification and regression tree. A binary tree was built similarly to a tree structure by splitting records according to a single input field at each node [27].

Each classification for SPCs was evaluated based on the area under the curve (AUC) of the receiver operating characteristic curve (ROC), which can also be used to determine how well a risk prediction model differentiates between patients with and without a certain condition. In general, the better the model discriminates, the closer the ROC curve approaches the upper left corner of the plot. In this study, there were 10 independent variables, generating 2^16^ input combinations, each of which yielded a predicted value, and the threshold value in the plot of the ROC curve was the result of the corresponding sensitivity and 1-specificity. For the risk factor rankings, GainRatio, InfoGain, RF, C5.0, and MARS classifications were selected. The ranking of each risk factor was determined by calculating the average ranking of the above methods. The final model performance was calculated by averaging the 10 classification accuracy metric results. These classifiers were modeled using “raprt”, “RWeka”, “mass”, “elmNN”, “e1071”, “lgr”, and “randomForest”, respectively, in the R environment, version 4.2.1. InfoGain and GainRatio were used with the Waikato Environment for Knowledge Analysis (WEKA), version 3.8. In the statistical testing methods, the independent variables included sex, age at diagnosis, tumor size, combined stage, group, radiotherapy, chemotherapy, body mass index (BMI) (kg/m^2^), smoking behavior, drinking behavior, and carcinoembryonic antigen (CEA) lab value. A *t*-test was used to compare SPCs and non-SPCs. We employed the chi-square test and odds ratio to assess the associations between the dependent variable and all independent variables.

In the clinical risk assessment, different decision tree models were used to identify the prediction factors of conditions of interest, namely support vector machines, linear discriminant analyses, logistic regression, C4.5 decision trees, classification and regression trees, random forests, and C5.0 decision trees. All subjects were divided into 10 subgroups, from the root to the leaf node, through different branches. By using these different decision tree models, clinicians can identify the combination of risk factors for the condition.

## 3. Results 

The descriptive characteristics of the study cohort are shown in Table 1. Of the 4402 patients in this study, 395 subsequently developed SPCs (males, 69.9%; females, 30.1%). The most frequent SPCs were colorectal (*n* = 231; 58.5%), followed by lung cancer (*n* = 42; 10.6%), others (*n* = 20; 5.1%), urinary system (*n* = 20; 5.1%), liver (*n* = 13; 3.3%), breast (*n* = 12; 3.0%), and prostate (*n* = 11; 2.8%). Our statistical analysis (see Table 1) indicated that sex, age at diagnosis, combined stage group, radiotherapy, chemotherapy, BMI, and smoking/drinking behavior revealed significant differences between rectal cancer patients who developed SPCs and those who did not. 

Table 2 depicts the ranking results of the importance of the 10 predictor variables derived from the GainRatio, InfoGain, RF, C5.0, and MARS models. The table shows the different classifications of the predictor variables by different classifiers. In addition, it shows the application of the Borda count procedure to combine the classification results and create a global ranking. In particular, chemotherapy appears to be a major risk factor among the treatments associated with SPCs.

**Figure 1:**
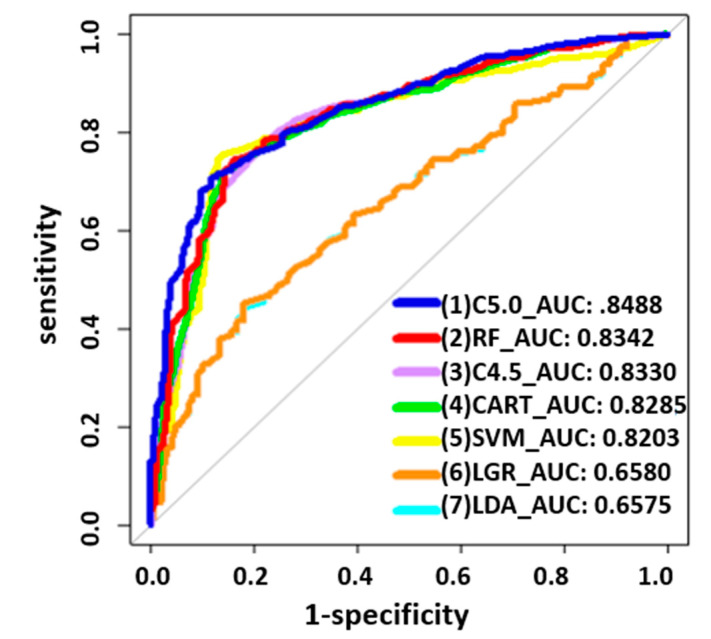
Receiver operating characteristic curves of the seven methods with AUCs for rectal patients receiving chemotherapy.

**Figure 2:**
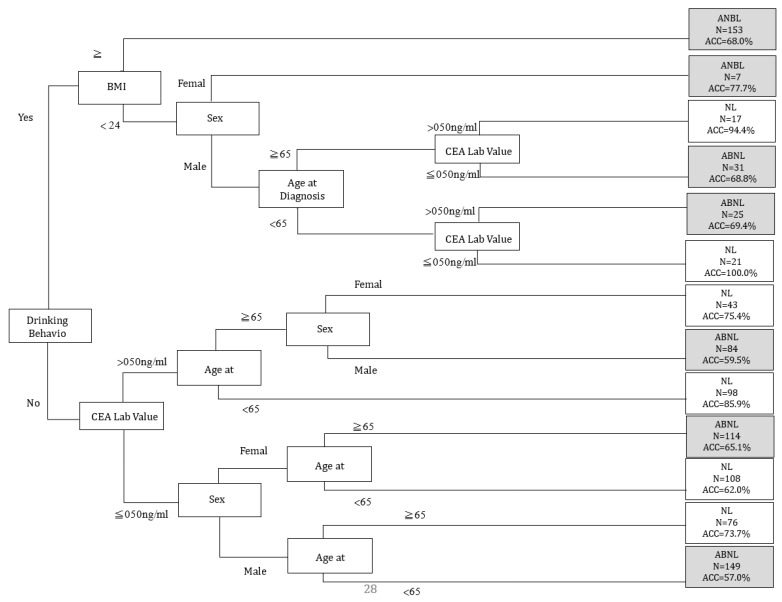
C5.0 classification tree depicting the secondary primary cancers (SPCs) of primary rectal cancer treated with chemotherapy. CEA Lab Value: carcinoembryonic antigen lab value; ABNL: abnormal; NL: normal; BMI: body mass index; ACC: accuracy.

For the variables with the highest AUC values, C5.0 showed more stable AUC performance (0.8488) than other classifiers. In order to further analyze the SPC predictor for the prediction of chemotherapy, we chose C5.0 as the basis for further analysis (see Figure 1, Table 3). To further analyze the risks associated with the occurrence of SPCs in rectal cancer patients after chemotherapy, we conducted demographic analyses of patients with chemotherapy, as shown in Table 4. Considering patients receiving chemotherapy, age at diagnosis (≥65 years; *p* = 0.004), smoking behavior (yes; *p* = 0.017), and drinking behavior (yes; *p* = 0.014) were associated with an increased risk of SPCs among patients with rectal cancer (see Table 4). Decision tree stratification based on C5.0 prioritized all independent variables to determine their branch status. Through different branches from the root node to the leaf node, all subjects were divided into 13 subgroups (see Figure 2). In the classified decision tree, drinking behavior was identified as the root node due to its strong influence on SPCs among rectal cancer patients. The following are the explanations of the relevant decision-making rules: The factors that determined the first rule decision tree were drinking behavior (no), CEA lab value (≤050 ng/mL), sex (male), and age at diagnosis (<65 years), resulting in an accuracy of 57.0% across 149 samples. A four-rule decision tree was developed based on drinking behavior (no), CEA lab value (≤050 ng/mL), sex (female), and age at diagnosis (≥65 years), resulting in an accuracy of 65.1% across 114 samples. The factors that determined the six-rule decision tree were drinking behavior (no), CEA lab value (>50 ng/mL), age at diagnosis (≥65 years), and sex (male), yielding an accuracy of 59.5% across 84 samples. Nine-rule decision trees were obtained considering drinking behavior (yes), behavior (yes), BMI (<24), sex (male), age at diagnosis (<65 years), and CEA lab value (>50 ng/mL), yielding an accuracy of 69.4% in 25 samples. We developed the 10-rule decision tree based on drinking behavior (yes), BMI (<24), sex (male), age at diagnosis (≥65 years), and CEA lab value (50 ng/mL), resulting in a precision of 68.8% in 31 samples. To create the 12-rule decision tree, we considered drinking behavior (yes), BMI (<24), and sex (female), yielding an accuracy of 77.7% in seven samples. The factors determined using the 13-rule decision tree were drinking behavior (yes) and BMI (≥24), yielding an accuracy of 68.0% in 153 samples. The rules related to the prediction models for SPCs in rectal cancer receiving chemotherapy are summarized in Table 5. 

## 4. Discussion 

SPCs are more likely to occur following improved survival in patients with rectal cancer. In this study, we observed that SPCs occurred in 395 (9.0%) of the 4402 primary rectal cancer patients. Of the treatments used for primary rectal cancer, chemotherapy posed the highest risk for developing SPCs. Considering patients receiving chemotherapy, the age of 65 years, smoking, and drinking behavior were strongly correlated with the development of SPCs in patients with rectal cancer. These findings provide important information for the effective prevention and surveillance of SPCs in rectal cancer survivors. This study reports several interesting findings. First, 9.9% of male and 6.4% of female survivors experienced SPCs during their follow-up. Zhang et al. reported a higher incidence of SPCs (males, 17.1%; females, 13.0%) in their colorectal study cohort [28]. Rectal cancer survivors, however, had an 8% higher rate of SPCs than the general population [29]. Our regression analysis indicated that sex, age at diagnosis, combined stage group, radiotherapy, chemotherapy, BMI, and smoking/drinking behavior were related to rectal cancer patients developing SPCs. As a result, it is important to consider the characteristics of cancer survivors, since these characteristics may influence their health in the future.

Most cancer survivors are increasingly concerned about the identification of the factors that may increase their risk of developing SPCs. Compared to radiotherapy, the use of chemotherapy was a more significant risk factor for the SPCs investigated here (see Table 1). As we know, many effective chemotherapeutic agents have recently been developed for the management of recurrence or metastases in rectal cancer [30,31]. Thus, carcinogenesis caused by increased use of these chemotherapeutic agents should be investigated. Similarly, Hung et al. reported that chemotherapy was significantly associated with all types of SPCs during the follow-up period in some cancer survivors [32]. However, although this study used cancer registry databases in several hospitals, no information was found about regimens of chemotherapy. Thus, utilizing different databases that include chemotherapy regimens is still necessary to validate our findings. 

The occurrence of SPCs is often recognized as a late adverse effect after cancer treatment. Since the risk of SPCs is not increased in the short term, long-term follow-up considering a latency period is necessary to observe this phenomenon. However, the risk pattern for SPCs has rarely been studied in depth; thus, this motivated us to perform the current analysis. According to our cancer registry database principles, our colorectal cancer patients treated with curative intent are routinely followed for 5 years. Our study materials complement existing data involving large populations and provide a more adequate duration of follow-up for assessing such low-frequency events. Although our results provide important insights into SPCs after rectal cancer treatments, with the increasing complexity of rectal cancer treatment, adding more information about modern techniques and drugs will be our next step. 

Radiotherapy is a part of the current standard treatment for rectal cancer. Radiation for tumor control causes early and late toxicity, which is associated with the subsequent development of SPCs [33]. A study conducted by Rombouts et al. using data from the Netherlands Cancer Registry from 1989 to 2007 found that patients who underwent RT for previous pelvic cancer were at greater risk of rectal cancer (subhazard ratio, 1.72; 95% CI, 1.55–1.91) [34]. After primary pelvic radiotherapy, another systematic review and meta-analysis found a small increase in the incidence of second primary cancer. However, since the introduction of modern radiation techniques, which provide excellent preservation of normal tissue, studies have shown that, in some cases, radiotherapy does not increase the risk of SPCs and might even have a preventive effect [35,36]. Therefore, a reliable and accurate method such as machine learning, which can take into account complex interactions between multiple predictor variables, can help to resolve this important question. 

Older age can lead to immunosenescence in survivors of SPCs, making it a critical prognostic factor [37]. SPC risk was determined by a combination of demographic factors, including age, race, and marital status, according to Zhang et al. [28]. Predisposition to a lifestyle such as smoking or alcohol also increases the risk of SPCs in cancer patients, particularly with respect to SPCs of the head and neck, esophagus, lung, urinary bladder, and kidney. Smoking and alcohol use cause damage to DNA damage repair in cells, and the length of exposure time increases the cancer risk. All this evidence supports our findings. In the study of the critical risk factors of secondary cancer in the medical practice, our results are consistent with existing research [15], showing that the C5.0 classification has greater compliance with clinical interpretations than alternative classification methods.

Postcancer treatment surveillance is crucial to detecting second lesions and improving survival. Of the treatments used for primary rectal cancer, chemotherapy posed the highest risk for developing SPCs in our results. An age of over 65, smoking, and drinking behavior are independent risk factors for SPCs after chemotherapy. These findings may help develop effective prevention and surveillance programs for high-risk rectal cancer survivors in their follow-up. For example, enhancing clinical health education on smoking cessation for elderly rectal patients is a recommended strategy. The government can also reduce the future occurrence of secondary cancers and subsequent treatment costs through smoking cessation policies.

This study has a few noteworthy limitations. First, data for our cohort were missing regarding dietary habits, comorbidities, and hereditary syndromes, which may significantly increase the risk of developing malignancies. Second, data regarding the type, length, and cycles of chemical agents administered were not available. However, we focused our analysis on the patients who received chemotherapy to increase the credibility of this study. Including the above variables in the model would help to extend the prediction performance. Third, the risk of SPCs varies according to race and ethnicity. Taiwanese residents, who are mostly Asian, accounted for 99% of our study cohort. Thus, our prediction models still need to be verified in external populations, although internal validation showed good consistency. Finally, our study used multiple machine learning models without comprehensive clinical validation, which may lead to overfitting and overly optimistic performance estimates. To address this, collecting new and unseen data for further validation is crucial. Additionally, the absence of detailed analyses may limit the ability to gain further insights. For example, future research should consider applying multiple testing adjustments, such as the Benjamini–Hochberg procedure [38], to reduce inflated false-positive rates. Moreover, incorporating calibration analysis, such as the Platt Scaling technique [39], is essential for future implementations to adjust predicted probabilities to more closely reflect actual outcomes, thus enhancing the model’s predictive accuracy.

## 5. Conclusions

Although patients with rectal cancer are at a high risk of developing cancer, current clinical guidelines do not include priority treatment strategies. This has resulted in significant changes in the quality of care provided to this population. As rectal cancer burdens continue to increase, it is important to evaluate current treatment strategy recommendations. An age of over 65, smoking, and drinking behavior are independent risk factors for SPCs after chemotherapy. These findings may help develop effective prevention and surveillance programs for high-risk rectal cancer survivors in their follow-up. This study aimed to perform a longitudinal diagnosis and prediction of SPCs among patients with rectal cancer. In addition to reassessing the risk factors for rectal cancer patients, the proposed model can also help to assess chemoradiotherapy response, particularly with the development of nonsurgical approaches such as “observation and waiting”. We suggest that future research further explore the relationship between the risk factors identified in this study. This study also serves as the basis for further clinical validation and a reference for healthcare education for both doctors and patients in the future.

## Figures and Tables

**Table 1:** Subject demographics of primary rectal cancer patients.

Risk Factors	With SPCs (%)	Without SPCs (%)	*p*-Value	χ^2^	Odds Ratio
*n* (%)	395 (9.0%)	4007 (91.0%)			
Sex	395	4007	0.004 *	8.477	
Male	276 (69.9%)	2503 (62.5%)			1.394 * [1.114–1.744]
Female	119 (30.1%)	1504 (37.5%)			1.00
Age at Diagnosis	395	4007	<0.001 ***	25.530	
<65 years	174 (44.1%)	2295 (57.3%)			1.00
≥65 years	221 (55.9%)	1712 (42.7%)			1.703 * [1.383–2.097]
Tumor size	395	4007	0.433	0.614	
<5 cm	267 (67.6%)	2630 (65.6%)			1.092 [0.876–1.362]
≥5 cm	128 (32.4%)	1377 (34.4%)			1.00
Combine Stage Group	395	4007	0.001 **	11.942	
≤stage II	212 (53.7%)	1787 (44.6%)			1.439 * [1.170–1.771]
>stage II	183 (46.3%)	2220 (55.4%)			1.00
Radiotherapy	395	4007	0.013 *	6.208	
No	279 (70.6%)	2579 (64.4%)			1.332 * [1.062–1.669]
Yes	116 (29.4%)	1428 (35.6%)			1.00
Chemotherapy	395	4007	<0.001 ***	20.410	
No	190 (48.1%)	1465 (36.6%)			1.608 * [1.307–1.979]
Yes	205 (51.9%)	2542 (63.4%)			1.00
BMI	395	4007	0.019 *	5.474	
<24	179 (45.3%)	2063 (51.5%)			1.00
≥24	216 (54.7%)	1944 (48.5%)			1.281 * [1.041–1.576]
Smoking Behavior	395	4007	<0.001 ***	12.882	
No	229 (58.0%)	2682 (66.9%)			1.00
Yes	166 (42.0%)	1325 (33.1%)			1.467 * [1.189–1.811]
Drinking Behavior	395	4007	0.001 **	10.224	
No	272 (68.9%)	3050 (76.1%)			1.00
Yes	123 (31.1%)	957 (23.9%)			1.441 * [1.151–1.805]
Carcinoembryonic Antigen (CEA) lab value	395	4007	0.894	0.018	
≤050	265 (67.1%)	2675 (66.8%)			1.015 [0.815–1.265]
>051–100, 987	130 (32.9%)	1332 (33.2%)			1.00

* *p* < 0.05, ** *p* < 0.01, *** *p* < 0.001.

**Table 2:** The relative importance of variables associated with SPCs in rectal cancer patients.

Rank	GainRatio *	InfoGain *	RF	C5.0	MARS	Overall
1	Age at Diagnosis	Age at Diagnosis	Age at Diagnosis	Sex	Age at Diagnosis	Age at Diagnosis
2	Chemotherapy	Chemotherapy	Chemotherapy	Radiotherapy	Smoking Behavior	Chemotherapy
3	Smoking Behavior	Smoking Behavior	Smoking Behavior	Drinking Behavior	Chemotherapy	Smoking Behavior
4	Drinking Behavior	Sex	Sex	Combine stage group	Tumor Size	Combine Stage Group
5	Sex	Drinking Behavior	Drinking Behavior	Chemotherapy	Combine Stage Group	Sex
6	Combine Stage Group	Combine Stage Group	Combine Stage Group	Age at Diagnosis	Radiotherapy	Drinking Behavior
7	BMI	BMI	BMI	Tumor Size	BMI	Radiotherapy
8	Radiotherapy	Radiotherapy	Radiotherapy	BMI	Carcinoembryonic Antigen (CEA) Lab Value	BMI
9	Tumor Size	Tumor Size	Tumor Size	Carcinoembryonic Antigen (CEA) Lab Value	Drinking Behavior	Tumor Size
10	Carcinoembryonic Antigen (CEA) Lab Value	Carcinoembryonic Antigen (CEA) Lab Value	Carcinoembryonic Antigen (CEA) Lab Value	Smoking Behavior	Sex	Carcinoembryonic Antigen (CEA) Lab Value

* Abbreviations: GainRatio, information gain ratio is the ratio of information gain to the intrinsic information. InfoGain, information gain is created by not providing a numerical difference between attributes with high distinct values from those that have less. GainRatio and InfoGain were obtained using the Waikato Environment for Knowledge Analysis (WEKA).

**Table 3:** Classification results of the rectal patients treated with chemotherapy.

Method	Specificity	Sensitivity	Accuracy	F1 Score	Precision(PPV)	NPV	AUC
C5.0	0.8812	0.7082	0.7941	0.7759	0.8579	0.9684	0.8488
RF	0.8377	0.7439	0.7905	0.7814	0.8228	0.9707	0.8342
C4.5	0.7565	0.7997	0.7783	0.7840	0.7689	0.9746	0.8330
CART	0.8551	0.7225	0.7883	0.7745	0.8347	0.9690	0.8285
SVM	0.8623	0.7554	0.8085	0.7988	0.8475	0.9728	0.8203
LGR	0.8188	0.4521	0.6343	0.5544	0.7166	0.9381	0.6580
LDA	0.8188	0.4421	0.6292	0.5455	0.7120	0.9371	0.6575

Abbreviations: FPR: the false-positive rate is the probability of incorrectly rejecting the null hypothesis for a particular test; MCC: Matthews correlation coefficient; a higher score is only obtained if the prediction had good results in all four categories of the confusion matrix (true positives, false negatives, true negatives, and false positives); F1 score: a harmonic score between sensitivity and precision; PPV: positive predictive value; NPV: negative predictive value.

**Table 4:** Subject demographics of primary rectal cancer patients with chemotherapy.

Risk Factors	With SPCs (%)	Without SPCs (%)	*p*-Value	χ^2^	Odds Ratio
*n* (%)	205 (7.5%)	2542 (92.5%)			
Sex	205	2542	0.1	2.702	
Male	143 (69.8%)	1628 (64.0%)			1.295 [0.951–1.763]
Female	62 (30.2%)	914 (36.0%)			1.00
Age at Diagnosis	205	2542	0.004 **	8.207	
<65 years	107 (52.2%)	1584 (62.3%)			1.00
≥65 years	98 (47.8%)	958 (37.7%)			1.514 * [1.138–2.015]
Tumor size	205	2542	0.831	0.046	
<5 cm	119 (58.0%)	1495 (58.8%)			1.00
≥5 cm	86 (42.0%)	1047 (41.2%)			1.032 [0.773–1.377]
Combine Stage Group	205	2542	0.397	0.718	
≤stage II	52 (25.4%)	579 (22.8%)			1.152 [0.830–1.600]
>stage II	153 (74.6%)	1963 (77.2%)			1.00
Radiotherapy	205	2542	0.302	1.065	
No	108 (52.7%)	1244 (48.9%)			1.162 [0.874–1.545]
Yes	97 (47.3%)	1298 (51.1%)			1.00
BMI	205	2542	0.382	0.764	
<24	101 (49.3%)	1333 (52.4%)			1.00
≥24	104 (50.7%)	1209 (47.6%)			1.135 [0.854–1.509]
Smoking Behavior	205	2542	0.017 *	5.695	
No	113 (55.1%)	1614 (63.5%)			1.00
Yes	92 (44.9%)	928 (36.5%)			1.416 * [1.063–1.886]
Drinking Behavior	205	2542	0.014 *	6.064	
No	134 (65.4%)	1863 (73.3%)			1.00
Yes	71 (34.6%)	679 (26.7%)			1.457 * [1.078–1.968]
Carcinoembryonic Antigen (CEA) Lab Value	205	2542	0.475	0.511	
≤050	115 (56.1%)	1491 (58.7%)			1.00
>051–100, 987	90 (43.9%)	1051 (41.3%)			1.110 [0.833–1.479]

* *p* < 0.05, ** *p* < 0.01.

**Table 5:** Summarized rules of condition risk factors.

Rules No.	Combinations of Condition Factors	SPCs/Observed (*n*)	Accuracy
**1**	Drinking Behavior (No) + CEA Lab Value (≤050 ng/mL) + Sex (Male) + Age at Diagnosis (<65 years)	149/261	57.0%
**4**	Drinking Behavior (No) + CEA Lab Value (≤050 ng/mL) + Sex (Female) + Age at Diagnosis (≥65 years)	114/175	65.1%
**6**	Drinking Behavior (No) + CEA Lab Value (>050 ng/mL) + Age at Diagnosis (≥65 years) + Sex (Male)	84/141	59.5%
**9**	Drinking Behavior (Yes) + BMI (<24) + Sex (Male) + Age at Diagnosis (<65 years) + CEA Lab Value (>050 ng/mL)	25/36	69.4%
**10**	Drinking Behavior (Yes) + BMI (<24) + Sex (Male) + Age at Diagnosis (≥65 years) + CEA Lab Value (≤050 ng/mL)	31/45	68.8%
**12**	Drinking Behavior (Yes) + BMI (<24) + Sex (Female)	7/9	77.7%
**13**	Drinking Behavior (Yes) + BMI (≥24)	153/225	68.0%

## Data Availability

Clinicopathological datasets are available from the corresponding author upon reasonable request.
